# A Proposal for an Individualized Pharmacogenetic-Guided Warfarin Dosage Regimen for Puerto Rican Patients Commencing Anticoagulation Therapy

**DOI:** 10.4172/2153-0645.T-001

**Published:** 2014-01-25

**Authors:** Luis Ángel Bermúdez Bosch

**Affiliations:** Pharmaceutical Sciences Department, Medical Sciences Campus, University of Puerto Rico, USA

## Abstract

Warfarin is the current standard of care in oral anticoagulation therapy. It is commonly prescribed to treat venous thromboembolism, pulmonary embolism, acute myocardial infarction, and to decrease the risk of stroke in atrial fibrillation. Warfarin therapy is challenging because of marked and often unpredictable inter-individual dosing variations that effectively reach and maintain adequate anticoagulation. Several researchers have developed pharmacogenetic-guided maintenance dose algorithms that incorporate genetics and individual patient characteristics. However, there is limited information available concerning dosing during warfarin initiation. This is considered the most clinically challenging therapeutic phase. In such, the risk of recurrent thromboembolism and hemorrhage are elevated. The objective of this retrospective study is to predict the individual initial doses for Puerto Rican patients (n=175) commencing anticoagulation therapy at Veterans Affairs Caribbean Healthcare System (VACHS) using pharmacogenetic/pharmacokinetic-driven model. A pharmacogenetic driven model (R2=0.4809) was developed in Puerto Rican patients and combined with pharmacokinetic formulas that enabled us to predict the individual initial doses for patients (n=121) commencing anticoagulation therapy. WinNonlin® pharmacokinetic-pharmacodynamic simulations were carried out to determine the predictability of this model. This model demonstrated promising results with few (n=10) simulations outside of their respective therapy range. A customized pharmacogenetic-based warfarin maintenance dose algorithm (R2=0.7659) was developed in a derivation cohort of 131 patients. The predictability of this developed pharmacogenetic algorithm was compared with the International Warfarin Pharmacogenomics Consortium (IWPC) algorithm and it demonstrated superior predictability within our study population.

## Introduction

Warfarin is the current standard of care in oral anticoagulation therapy [1]. The treatment indications for warfarin use include; venous thromboembolism, pulmonary embolism, acute myocardial infarction, and to decrease the risk of stroke in atrial fibrillation [2]. Although its efficacy and safety has been compared with new anticoagulation medications [2], warfarin continues to be the standard choice in anticoagulation therapy. Its initial approval remotes back to 1954 [3]. Since then, for almost 60 years, it has remained the most widely prescribed oral anticoagulant drug. In 2010, more than 23 million prescriptions were documented in the United States [4]. Warfarin occupies the 11^th^ place in drug sales in the United States [5] but 2^nd^ place in adverse effect reports [6] in outpatients. These adverse effects can be attributed to the challenging and often unpredictable inter-individual dosing variation that effectively reach and maintain adequate anticoagulation. For most patients, ideal therapy is accomplished by maintaining the international normalized ratio (INR) within a therapeutic range of 2.0–3.0. Incorrect warfarin doses can lead to insufficient antithrombotic effect, or over-anticoagulation that might expose patients to elevated bleeding risk [7]. The most common advantages and disadvantages of warfarin therapy are summarized in Table 1.

**Figure 1:**
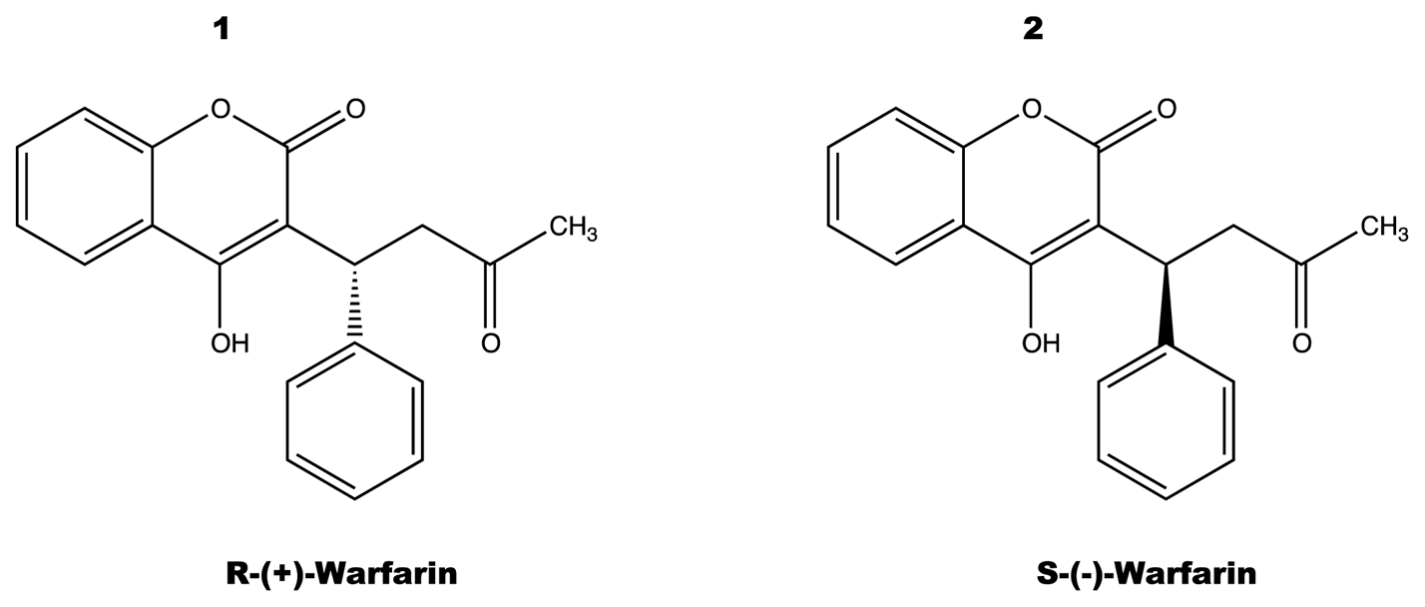
Warfarin structures were drawn using CS ChemDraw Ultra^©^ v.12.

Warfarin is supplied as a racemic mixture of enantiomers R and S (Figure 1) [1]. Studies have demonstrated that the S-enantiomer exhibits 3 to 5 times more anticoagulant activity than the R-enantiomer, but generally has a more rapid clearance [8]. The half-life of R-warfarin is 45 hours while that of S-warfarin is 29 hours. As a racemic mixture, the half-life of Warfarin ranges from 36 to 42 hours [9].

Warfarin is principally stereo-selectively metabolized by hepatic cytochrome P-450 (*CYP450*) into metabolites with minimal anticoagulant activity [10,11]. *CYP2C9* is the primary enzyme responsible for metabolism of the active *S-*enantiomer of warfarin [7]. One particular study [12] demonstrated significant involvement of *CYP2C19* upon warfarin’s action and metabolism. Identified metabolites include; dehydrowarfarin, two diastereoisomer alcohols, and 4′-, 6-, 7-, 8-, and 10-hydroxywarfarin [10].

**Figure 2:**
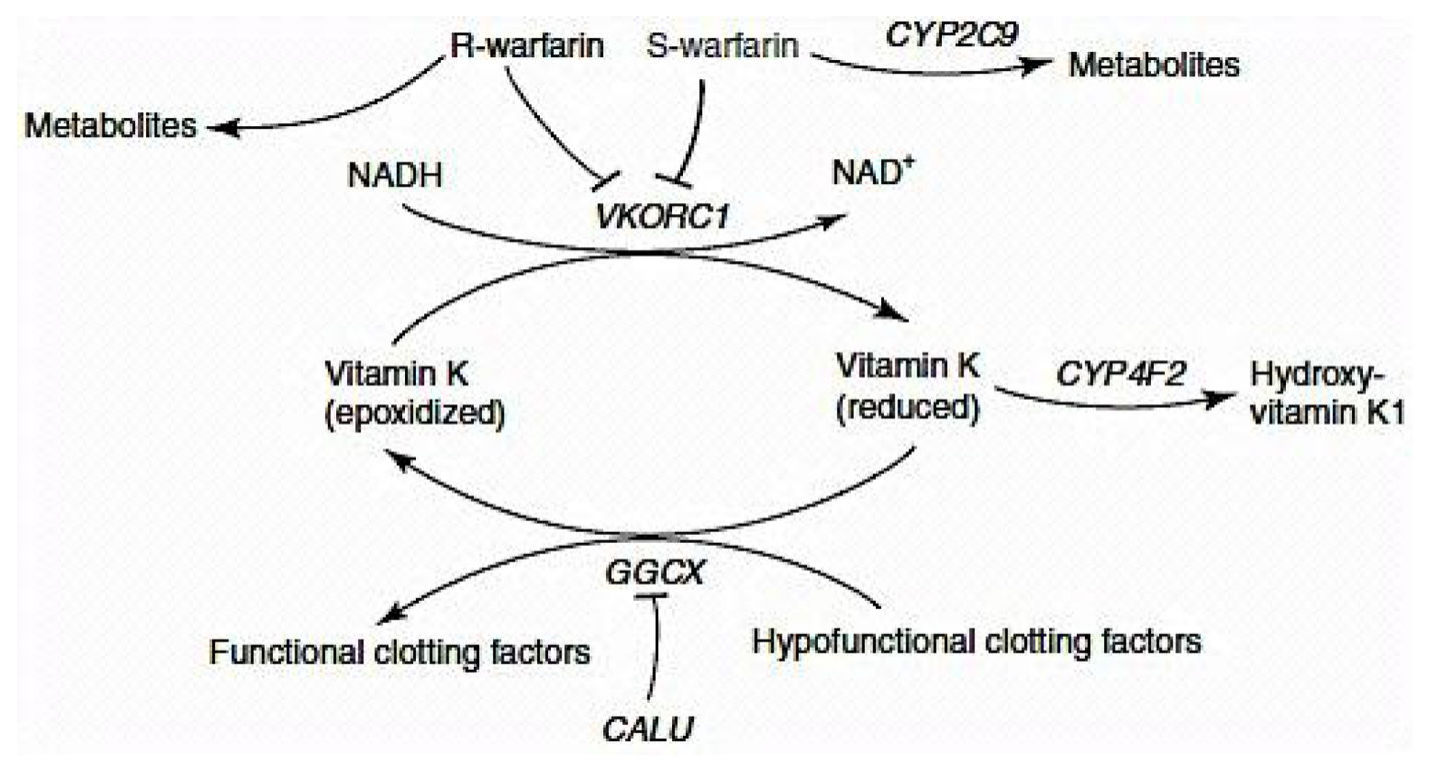
Metabolism and Mechanism of Action of Warfarin [1]. Image was used with Nature Publishing Group authorization. Nature Publishing Group license Agreement #3199600168134.

Warfarin acts by inhibiting anticoagulant proteins C and S [13], and by inhibiting the synthesis of vitamin K-dependent clotting factors, these include Factors II, VII, IX, and X [14]. It is believed that warfarin’s inhibition of the C1 subunit of vitamin K epoxide reductase (*VKORC1*) enzyme complex interferes with clotting factor synthesis, which enables it to reduce the regeneration of vitamin K1 epoxide [13]. The final effect in this chain is the desired therapeutic effect, anticoagulation (Figure 2).

Warfarin therapy is very challenging due to its narrow therapeutic index and great inter-individual variability in patient response. As a result, warfarin is a leading cause of serious medication-related adverse events, and its efficacy is also suboptimal [1,15]. In 2007, the US Food and Drug Administration (FDA) updated the label of warfarin to note the importance of *VKORC1* and *CYP2C9* polymorphisms, which have been confirmed to contribute significantly to the variability in warfarin dose requirements [16]. *VKORC1* and *CYP2C9* are involved in warfarin pharmacokinetics and pharmacodynamics.

*VKORC1* polymorphism explains 30% of the dose variation between patients [17]: mutations make *VKORC1* less susceptible to suppression by warfarin [18]. The importance of this gene is vital because *VKORC1* is the enzyme that regulates coagulation via redox reactions upon vitamin K where the oxidized form of vitamin K will lead to the production of functional prothrombine and other coagulation dependent factors; while the reduced form will lead to hypofunctional coagulation factors and prothrombine. One remarkable study highlights *VKORC1* importance during warfarin initiation phase [18]. It must be point out that recent studies have reported that the allele *CYP4F2* is involved in vitamin K metabolism, and polymorphisms of *CYP4F2* can affect Vitamin K oxidase activity [10,19,20]. Some studies suggest that patients with *CYP4F2* are most likely to require higher doses to achieve an anticoagulation response [19,21].

The *CYP2C9* gene is of particular interest because it exhibits marked genetic polymorphisms. Polymorphisms in *CYP2C9* contribute significantly to variability in warfarin response [12,18,20,22]. The highly polymorphic *CYP2C9* gene has 35 known variant alleles [23], many of which result in decreased enzyme activity [24,25]. The results of this can affect the activity of such by increasing, decreasing, or even inactivating it completely. CYP2C9*1 is the wild type polymorphism which promotes normal metabolism of warfarin S-enantiomer [18]. The most common variants, CYP2C9*2 and CYP2C9*3, exert less effect upon warfarin metabolism than CYP2C9*1 [18]. Various studies have demonstrated that the presence of CYP2C9*2 and CYP2C9*3 result in a respective decrease of 15–20% and 30–40% of the stable warfarin dose requirements when compared to the wild-type CYP2C9*1 [18,24]. CYP2C9*5 and CYP2C9*6 alleles are found almost exclusively in African Americas and have been associated with reduced enzyme activity and reduced clearance of *CYP2C9* substrates [19]. The CYP2C9*8 allele, is almost twice as common as the CYP2C9*2 and CYP2C9*3 alleles combined in African Americans [22]. This allele also requires significantly lower warfarin doses to achieve optimal anticoagulation as compared to CYP2C9*1 allele [22]. In the development of this algorithm, it was taken into consideration polymorphisms up to CYP2C9*6 because their activity have demonstrated greater effects on the anticoagulation status than the rest of the polymorphisms [1,18,25,26].

Several researchers have developed pharmacogenetic-guided maintenance dose (Md) algorithms that incorporate genetics and individual patient characteristics, such as age, weight, and body surface area (BSA) [24,27–32]. Only one noticeable investigation outside of our group was carried out on a Hispanic population which included 50 patients [33]. By incorporating the following variables: *CYP2C9* (*1, *2, *3) &*VKORC1* genotypes, age, BSA, venous thromboembolism, the researchers obtained an R^2^ of 56%. 89% of the sample population was of Mexican descent [33].

However, there is limited information available concerning dosing during warfarin initiation. This is considered the most clinically challenging therapeutic phase. In such, the risk of recurrent thromboembolism and hemorrhage are elevated [34–36]. In such phase, pharmacogenetic-based dosing has the potential to reduce these risks and improve the onset of a full therapeutic effect, which could potentially reduce inpatient hospitalization costs. Therefore, based on the need of an algorithm that takes into consideration genetic and individual patient characteristics from our Puerto Rican population, our investigation was defined according to several objectives and a central hypothesis.

## Objectives

- To predict the individual initial doses for patients commencing anticoagulation therapy using pharmacogenetic/ pharmacokinetic-driven model in Puerto Ricans.
- To develop a customized pharmacogenetic-based maintenance warfarin-dosing algorithm in a derivation cohort of Puerto Rican patients from the Veterans Affairs Caribbean Healthcare System (VACHS).

## Hypothesis

A personalized prescribing algorithm can be developed and validated *in silico* for a warfarin starting dosing, based on individual genotypes and INR response, to customize the treatment of Puerto Rican patients that initiate warfarin therapy.

## Materials and Methods

### Study population & clinical data collected at VACHS

**Figure 3:**
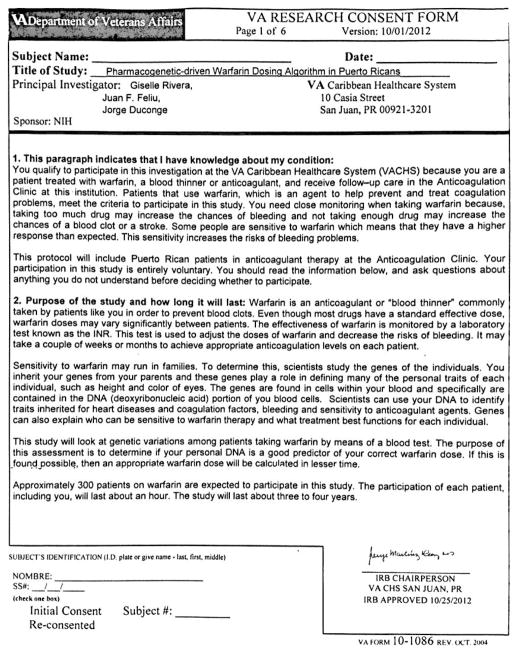
VACHS Research Consent form.

The study population consisted of 138 patients from the VACHS facilities located in San Juan, Puerto Rico. The requirement for warfarin therapy was determined on the basis of current American College of Chest Physicians guidelines [7]. Study eligibility was determined by the following inclusion criteria: (1) Puerto Rican Hispanic origin (both of the patient’s parents must be of Puerto Rican origin). (2) Age between 21 and 90 years. (3) Receiving warfarin for therapeutic anti-coagulation. (4) Stable warfarin dose with three target INR range of 2.0–3.0 or 2.5–3.5, according to indication such as deep vein thrombosis (DVT) with or without Pulmonary Embolism (PE), atrial fibrillation (AF) or other arrhythmias, cardiac valvular replacement, previously diagnosed coagulopathy. (5) Hematocrit (HCT) >40. Exclusion criteria included the following: (1) Non-Hispanic (race/ethnicity is self-reported by the patients). (2) Non-Puerto Rican origin patients (with at least one of his/her parents from other ethnicity group). (3) Currently enrolled in other active research protocols at the VACHS Hospital. (4) Blood urea nitrogen (BUN) >30 mg/dL. (5) Serum creatinine (sCr) >2.0 mg/ dL. (6) Active Hepatic disease (defined by a Child-Pugh score above 10 points: ascites; total bilirubin above 2.0 mg/dL; serum albumin below 3.5 g/dL. Prothrombin time in seconds prolonged over control >4; hepatic encephalopathy). (7) Prolonged diarrhea (three or more days). (8) Nasogastric or enteral feedings. (9) Acute illness (e.g. sepsis, infection, anemia, cancer). (10) LFT <3× ULN. (11) Active malignancy. (12) Known or suspected pregnancy. Patients who met the eligibility criteria were contacted on their next appointment at VACHS. A written consent form (Figure 3) was required for patients who voluntarily wished to participate in our investigation.

Blood collection for DNA typing: 2–3 ml EDTA blood sample per patient will be drawn in lavender/purple-colored stopper vacutainers® tube for analysis of the cytochrome P450 2C9 gene (*CYP2C9*) and Vitamin K epoxide receptor subunit-1 gene (*VKORC1*) alleles, at the time of routinely scheduled PT/INR testing collections. The tube will be gently inverted approximately 10 times to ensure proper mixing. The corresponding patient’s code number will be printed on the tube label.

### Confidentiality and privacy

**Figure 4:**
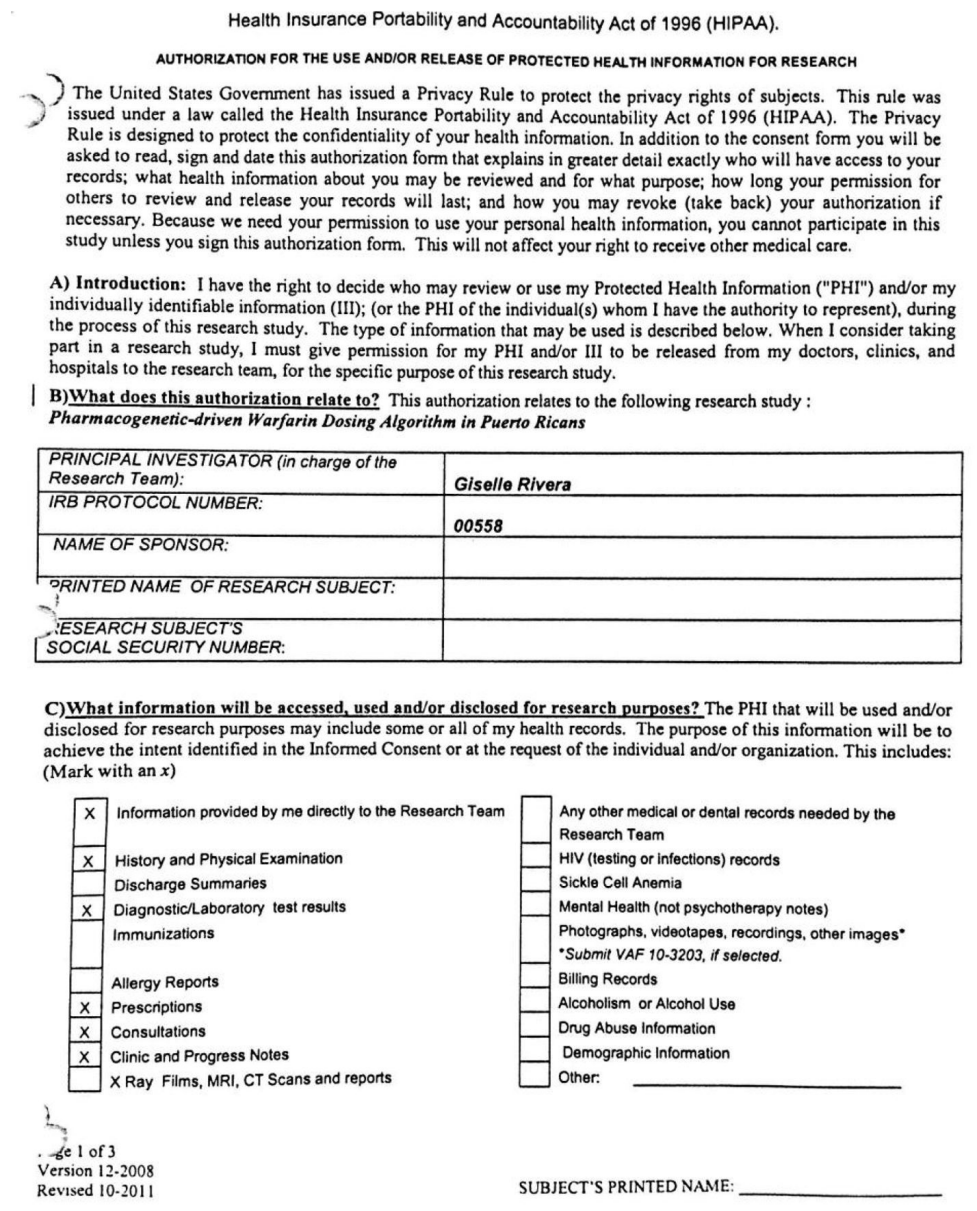
Health Insurance Portability Accountability Act (HIPAA) authorization form.

Appropriate safeguards against any potential violation of privacy and/or breach of confidentiality were ensured. Any information that could directly identify the participants, were kept safe by our researchers using an encryption method. Patient’s medical records were separated from the study database and codes. All these records reside electronically in the VA server (VACHS, San Juan facilities) as well as the files containing the codes for both blood and extracted DNA samples, which link identifiable personal information with the relevant research-related data obtained from the study. Codes were stored and encrypted in separate files from records and databases, and were only used to perform the necessary data assembly. This was executed by making each individual unique genotype (i.e., wild-type, single carrier, double carrier) and his/her related demographic/clinical covariates (e.g., weight, sex, gender) correspond to his/her stable warfarin dose and/or anticoagulation-related outcomes as specified in the protocol procedures. In doing so, the statistical analysis is an attempt to identify whether clinical correlations exist. Codes do not contain letters/ numbers that might link patients that participated in this study. After the completion of this research, the principal investigator (PI), a VA employee, (Dr. Giselle Rivera, PharmD) will store these master records/ files, study databases, and any other relevant documents related to study findings within the VACHS Research Office. Any other file containing relevant data collected from VACHS records used to perform any specialized analysis outside the VACHS facility will be deleted, following an accepted de-identification procedure (overwriting, degaussing, etc.). Once this procedure is performed, there will be no way to determine which specific sample the patient provided. All the specimens (i.e.: patients’ blood samples and extracted DNAs; including fractions for retrieval and/or replications) that were processed as part of this study were used for only this purpose. Therefore, the risk of any privacy violation was controlled. Individual blood samples were completely discarded at the moment of DNA extraction, following the current policy at UPR-Medical Sciences Campus for safety disposal of biohazard materials. De-identified DNA specimens will be stored frozen in eppendorf® tubes at the UPR-Medical Sciences Campus facility (Co-PI’s Laboratory A-640, RCMI Core Laboratory of Molecular Genetics, 6^th^ floor, main building) until the end of the study. For this purpose, a 7-digit code will be used to link each deoxyribonucleic acid (DNA) specimen with the corresponding personal identifiable information that will only reside on VA protected environment at the PI’s office. Collaborators in the study, and particularly the personnel at either the Laboratory of Personalized Health-Genetic Research Center (Hartford, CT) or the RCMI Molecular Genetic Core Lab (Medical Sciences Campus, University of Puerto Rico), do not have access to any information that identify the enrolled patients. All specimens will be destroyed and properly disposed after processed therein. Caution was taken to protect and avoid unnecessary disclosure of any research-related health information that arose during this study because such action is irrelevant for the purposes of this survey. The protected health information was not reused or disclosed to any other person or entity. The ethics committee overseeing the research done in VACHS reviewed the data obtained from this study. Personal (individual) identifiable information and/or any other relevant research-related health data from candidates was only collected, assembled and accessed by the research members of this study. The PIs and/or study coordinator were responsible for overseeing the security and confidentiality of such records. This information was mainly copied from patient’s medical records using Computerized Patient Record System (CPRS) after previously obtaining the corresponding signed authorizations from patients. For further statistical analysis, data (excluding patient name, medical record, or any other identifier that would reveal patient identity) was exported using encrypted language to secure information against eventual lost or theft. Raw data from genotyping analysis of each sample as well as details of the performed procedures/assays to call a genotype was recorded on the corresponding workbooks, which resides within the laboratory facilities as required by good laboratory practice and University of Puerto Rico - Medical Sciences Campus (UPR-MSC) policies. The raw data is entirely de-identified and therefore, risk of privacy or confidentiality breach does not exist. Information used and/or disclosed for research purposes: The following *Protected Health Information* (PHI) and/or *Individually Identifiable Information* (III) was collected/used from recruited patients and/or disclosed in order to achieve the research purposes of this study as described above: (1) information provided directly by the participant to the research team (e.g., by questionnaires); (2) information collected directly from the CPRS by the research team. Such information includes history and physical examination; diagnostic/laboratory test results; prescriptions; consultations; and clinic and progress notes. Although the PI and her research team will be the only ones allowed to use this information, they may share some patient’s PHI with the following parties in order to audit or monitor the quality and safety of the research activity: Institutional Review Boards, the Department of Health & Human Services or other United States government agencies, as required by law. In this study, every patient whom consents to participate will be required to sign a HIPAA authorization form (Figure 4) that will be valid until completion of this study.

### Whole blood sample collection, transfer, extraction & quantification

A small blood sample (approximately 5 ml) for DNA analysis was taken from each participant at the same time of their scheduled routinely INR measurement in the VACHS facilities. In order to minimize potential risk upon blood withdrawal, licensed phlebotomist of the VACHS will perform this procedure. No additional appointments and/or specimen collections will be scheduled. Blood samples in the containers will be fractionated into two to three portions for retrieval and replicate purposes. The sample collected from each patient will be stored in vacutainers® and will be immediately coded using a seven-digit unique study number by the study coordinator. This will be printed on each specimen tube label so that the sample will not directly identify the patient. No other labels or identification will be printed on the sample container. The samples will then be placed in plastic biohazard bags and then stored in a blood transfer cooler for transportation to the University of Puerto Rico Medical Sciences Campus laboratory. Containers will be placed on ice and stored frozen until genomic DNA extractions at the RCMI Core Laboratory (A-640, 6^th^ floor, Main Building, University of Puerto Rico, Medical Sciences Campus) can be performed.

The DNA was extracted and purified from the samples using the QIAGEN® QIAamp Blood Maxi kit by trained personnel. QIAamp DNA Blood technology yields genomic, mitochondrial, or viral DNA from blood. The procedure is briefly described as follows: Each sample will separately mix into the bottom of a centrifuge tube (50 mL); (500 μl) QIAGEN® Protease and the blood sample (5 mL) and mixed briefly. This step is followed by the addition of Buffer AL (6 mL) and each sample will be mixed thoroughly by vortexing at least 30 seconds. To ensure adequate lysis, the sample must me mixed toughly with Buffer AL to yield a homogenous solution. Note: Do not add QIAGEN Protease directly to Buffer AL. The samples will be incubated at 70°C for 10 min. DNA yield reaches a maximum yield after lysis for 10 min at 70°C, but longer incubus time will not adversely affect yield. After the samples cool down, add ethanol 99.5% (5 mL) to each sample and mix by vortexing as previously described. Note: Only use ethanol since other alcohols may result in reduced yield and purity. In order to ensure efficient binding, it is essential that the sample be mixed thoroughly after addition of ethanol to yield a homogeneous solution. Then, carefully apply half of the solution (50 mL) from the previous step onto the QIAamp Maxi column placed in a centrifugation tube (50 mL) which is provided in the QIAGEN® QIAamp Blood Maxi kit. Samples will be centrifuged at 3000 rpm for 3 min at 25°C. Following this procedure, the filtrates can be discarded, and Buffer AW1 (5 mL) will be placed in the QIAamp Maxi column. Samples will be centrifuged at the same conditions as before but at 4000 rpm. Then, proceed with the addition of Buffer AW2 (5 mL) to the QIAamp Maxi column and centrifuge at 4000 rpm for 15 min at 25°C. After this procedure, the centrifugation tube (50 mL) that contained the filtrate, was discarded and place the QIAamp Maxi column in a clean centrifugation tube (50 mL) which is provided with the kit. Note: Wipe any spillage off the QIAamp Maxi column before insertion into the clean 50 mL centrifugation tube. The samples are then incubated as previously described to evaporate any residual ethanol. Once the samples achieve room temperature, Buffer AE (1 mL) is added by pipetting it directly onto the membrane of the QIAamp Maxi column and then it will be centrifuged using previous conditions for 2 min. Following this procedure the eluted filtrate containing the DNA is reloaded onto the membrane of the QIAamp Maxi column and is centrifuged as before for 4 min. For long-term storage of DNA, eluting in Buffer AE and storing in aliquots at −20°C is recommended, because DNA is subjected to acid hydrolysis if dissolved in water. As a measure of quality control, 2 or 3 random samples will be analyzed for the presence of DNA, for every 10 samples by Polymerase Chain Reaction (PCR) (*As described in the next paragraph*). Finally, 150 μL of each sample in plastic tubes (1.5 mL) was transferred. These will also be identified with the seven-digit unique study number printed on the corresponding tube label. Each sample will be sealed with parafilm® paper, stored in plastic biohazard bags and frozen until assay. These samples will later be stored in a blood transfer cooler and be shipped to the Genomas Inc. in Hartford, Connecticut.

The extracted DNA was stored at −80°C in TRIS-EDTA buffer (TE). Quantification of DNA was performed by fluorescent staining of double stranded DNA (PicoGreen® dsDNA Quantitation Kit, Molecular Probes, Eugene, OR). Fluorescent intensity will be measured using a fluorescent micro-titer plate reader (POLARstar OPTIMA, BMG LABTECH GmbH, Offenburg, Germany). The concentration of extracted DNA will be adjusted to12.5 ng/μL in DNase free distilled water. A total of 25 ng of extracted DNA is required for the PCR reaction using the Tag-It™ Mutation Detection Kits from Tm Bioscience.

### DNA analysis & variables to be collected (*CYP2C9* & *VKORC1*) at the laboratory of personalized health (LPH), Hartford, Connecticut

Genomas Inc. in Hartford, Connecticut, performed DNA typing assays at the Laboratory of Personalized Health (LPH), using the HILOmet Warfarin system. The LPH, a division of Genomas Inc., is located at the Hartford Hospital Genetics Research Center in The Florence Crane Building, 67 Jefferson Street (Hartford, CT) and has been in operation since April 2005. LPH is a highly complex clinical DNA testing center licensed by the Connecticut Department of Health (CL- 0644) and certified by the Centers for Medicare and Medicaid Services (ID# 07D1036625) under CLIA (Clinical Laboratory Improvement Amendments). The HILOmet system employs a Luminex®100 analyzer using xMAP® technology (Luminex Corp., Austin, TX) installed at the LPH. The genotyping kits are from Tm Bioscience (Toronto, Ontario, Canada). The assay requires 50 ng genomic DNA. The assay *CYP2C9* and *VKORC1* variants detected though this assay are summarized in Table 2.

#### Genotypes

The most common alleles of the *CYP2C9* gene (*1 to *3) responsible for the metabolism of warfarin, and the two most common alleles for the *VKORC1* gene (G/A at −1639) responsible for the action of warfarin, will be the focus of this study [1,17,30]. However, our laboratory analysis will also include other important but less common allele variants. Other variables taken into consideration for analysis were: Demographic data including age, gender, height, weight, BMI, self-reported racial/ethnicity and other clinical data included; indication for warfarin therapy, comorbidities, concomitant medications, stable warfarin dose, target INR, initial INR, dose adjust INR, INR days 1 to 5 and genetic information [18,28–30]. All relevant non-genetic data was retrospectively obtained from CPRS and questionnaires. This data was recorded on a MS Excel formatted clinical database by the PI’s and staff.

### Dosage variability& statistical analysis

All patients with complete genetic and clinical data from the VACHS (n=138) were selected as the ‘derivation cohort’ for developing a pharmacogenetic/pharmacokinetic-driven initial warfarin dose prediction model in Puerto Ricans. A multiple linear regression analysis was performed using maintenance dose as the dependent variable, following a stepwise addition and backward elimination regression procedure to determine whether the mayor *CYP2C9* and *VKORC1* allele variants explained variability in stable warfarin dosage in this study population. Based on the partial correlations after considering the effects of genotypes, also it was considered age, BMI, BSA, sex, indication, comorbidities, concomitant medications, vitamin K intake, initial INR, target INR, dose adjust INR and INR days 1 to 5 as potential regressor variables that independently explain warfarin dose variation. Using this regression model, a warfarin-dose algorithm for the VACHS Puerto Rican population that predicts the best dose for stable anticoagulation was developed. Variables were included in the final regression model if they were significant (p<0.05), represented a biological plausibility (0.05 ≤ p ≤ 0.15), or if they were known clinical variables that affected warfarin dosage variability. We then combined our derived pharmacogenetic-based model with formulas by Avery et al. [37] to predict the individualized initial doses for patients commencing anticoagulation therapy. This enabled us to establish an initial dose regimen that not only predicted the effective warfarin maintenance doses (mg/day) in each single Puerto Rican patient (mainly in those at the highest risk of poor control), but also to predict the individualized initial doses for patients commencing anticoagulation therapy. Through the combination of our pharmacogenetic model with an estimated accumulation index that is based on differences in warfarin clearance due to *CYP2C9* genotypes, according to the formulas derived by Avery et al. [37].

$$d=\mathrm{IDA}\times \left[\frac{\frac{1}{\left(1-e^{-kt}\right)}-\left(1+e^{-kt}+e^{-k2t}\right)}{\frac{1}{3}+\frac{2e^{-kt}}{3}+e^{-k2t}}\right]$$

Where, IDA is the calculated maintenance dose per day in mg according to our pharmacogenetic-based model; k is the elimination rate constant of warfarin given a *CYP2C9* genotype: *1/*1=0.0189 hr^−1^; *1/*2=0.0158 hr^−1^; *1/*n=0.0132 hr^−1^; *2/*2=0.0130 hr^−1^; *2/*n=0.009 hr^−1^; *n/*n=0.0075 hr^−1^, being n=*3, *5 or *6. The starting doses of warfarin on days one to three are then calculated as:

$$\mathrm{day}\;\mathrm{one}=MD+d;\mathrm{day}\;\mathrm{two}=MD+\frac{\mathrm{2d}}{3};\mathrm{day}\;\mathrm{three}=MD+\frac{d}{3}.$$

Doses were divided to avoid an over shoot in INR response over desired therapy range. This method to predict such doses during the induction phase during days 1, 2 and 3 has been previously used in the EU-PACT trial [34]. Using the available data set, dose predictions from our pharmacogenetic-pharmacokinetic model were compared with actual doses using the Mean Absolute Error (MAE; mg/day). MAE is defined as the mean of the absolute values for the difference between the predicted and actual doses; it is used to evaluate the model’s predictive accuracy. The MAE was computed in the original units, rather than in the log-transformed units, to allow an impartial comparison of all models. The final model as the one that offered the lowest predictive MAE was selected. The bias of the dosing algorithm estimates (precision) was assessed by calculating the mean percentage of difference from the observed dose, where mean percentage of difference is equal to the MAE between predicted and actual dose, divided by the actual dose ([predicted dose - observed dose]/observed dose)×100%. Finally, the effect size of each independent predictor covariate on the log-transformed daily dose of warfarin was also computed.

### WinNonlin^®^PK-PD simulations

**Figure 5:**
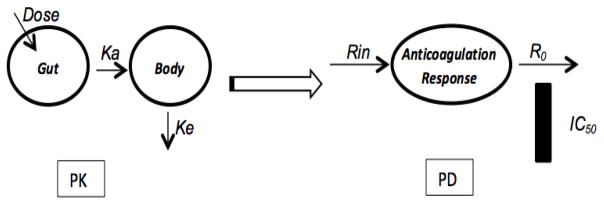
Schematic representation of the indirect pharmacokinetic-pharmacodynamic (PK-PD) model to be employed. Ka=Absorption rate constant, Ke=Elimination rate constant, Rin=Input rate, R0=Rate of elimination, IC50=half maximal inhibitory concentration, PK=Pharmacokinetic, PD=Pharmacodynamic. Parameter values used for simulation of warfarin levels and INR response time course during model development are shown in the Table 3.

The corresponding simulations of INR levels over first 10 days of treatment with warfarin were performed for each participant using a Jusko-type Indirect Response Model (IRM) for an inhibitory effect, based on their individual *CYP2C9* genotype data and population average parameters. Analyses were conducted through WinNonlin® (WinNonlin® professional software, version 2.1, Pharsight Inc., 1997, NC, USA). Pharmacokinetic (PK) of warfarin was described by a one-compartment model, with first-order absorption and linear elimination rate while the pharmacodynamic (PD) response was simulated by an indirect model that accounts for delay in anticoagulation response. A schematic representation of the indirect pharmacokinetic-pharmacodynamic (PK-PD) model to be employed in the simulation is depicted in Figure 5.

Initial warfarin doses (mg/day) used in the simulation procedures was determined by a previously developed pharmacogenetic-driven algorithm in Puerto Ricans. The average IC_50_ value for warfarin inhibition of Vitamin K recycling was set at 1.5 mg/L for all cases, which are the plasma warfarin concentration required to reach a 50% reduction in synthesis/activation of prothrombin-related, calcium-dependent clotting factors (i.e., factors II, VII, IX, X, protein Z and C) and a corresponding doubling of the INR level.

## Results

Our derivation cohort consisted of 175 enrolled patients. A total of 37 patients were removed for the following reasons; twenty-five patients were excluded because of lack of admixture index information; three samples were excluded because of poor genotyping call rate; two were excluded due to mild impaired decision-making capability and another seven individuals were removed due to lack of complete clinical data availability from CPRS. The population available for analysis consisted of 138 male patients. The mean age was 68 ± 9.2 years old. 89.9% (n=124) identified themselves as white. AF was the most common indication for warfarin use in 71.7% (n=99), followed by DVT in 19.6% (n=27) and PE in 5.8% (n=8). Diabetes was present in 28.3% (n=39) of our cohort. 7.25% (n=10) confirmed they smoke on a daily basis and 12.32% obtained a high source of vitamin K from their diet. Statins were the most common class of drugs co-administered among warfarin in 52.5% (n=64), followed by Amiodarone in 2.9% (n=4) of our study sample. The mean warfarin doses were the following; dose 1: 4.01 ± 0.12 mg/day, dose 2: 4.07 ± 0.12 mg/day, dose 3: 4.14 ± 0.12 mg/day. Mean INR measurements in patients with therapeutic range between 2–3 (n=129) were the following; day 1: 1.98 ± 0.10, day 2: 2.22 ± 0.09, day 3: 2.40 ± 0.10, day 4: 2.31 ± 0.07 and day 5: 2.31 ± 0.10. Only 9 patients had an INR target between 2.5–3.5. Their mean INR measurements were the following; day 1: 2.33 ± 0.41, day 2: 2.09 ± 0.30, day 3: 2.46 ± 0.56, day 4: 2.34 ± 0.26 and day 5: 2.52 ± 0.29. Average warfarin doses ranged from 0.86 to 8.00 mg/day. The mean initial warfarin dose was 4.01 ± 0.12 mg/ day. *CYP2C9* wild type polymorphism predominated in 73.9% (n=102) within this group, followed by single carriers with 19.6% (n=27) and double carriers with 6.5% (n=9). Polymorphism in *VKORC1* GA was the most common within our group with 45.7% and *VKORC1* GG with 41.3% and *VKORC1* AA were present at a lesser degree with 13.0%.

A multivariate least-squares linear regression model that predicts the log-transformed effective warfarin dose was developed to establish a pharmacogenetic-guided algorithm for warfarin therapy initiation (Equation 1). This model incorporated demographic, genetic and clinical variables, which demonstrated to be the best alternative for the available data, following the lowest mean absolute error criteria. For this least-squares linear regression analysis, an additional 17 participants where automatically excluded by Stata®v.12 software because of lack of information regarding stain use (n=16) and vitamin K intake (n=1). This regression model included 121 patients, the variables included in our algorithm were; age (p-value=0.003), BSA (p-value=0.406), *CYP2C9* (p-value<0.0001), VKORC1 AA (p-value=0.001), VKORC1 GA (p-value<0.0001), Admixture Index 2 (Taíno) (p-value=0.345), Admixture Index 3 (African) (p-value=0.192), Admixture Index 4 (Mixed) (p-value=0.119), Target INR (p-value=0.093), Statin use (p-value=0.415), Amiodarone use (p-value=0.168), Smoker (p-value=0.368), diabetes (p-value=0.040) and vitamin K intake (p-value=0.297). Overall, the pharmacogenetic model for our initiation dose regimen contained 12 variables. Of these, 5 were statistically significant (p<0.05), and 2 resented a biological plausibility (0.05 ≤ p ≤ 0.15). The regression analysis for this model is summarized in Table 4.

**Equation 1**: Initiation Dose Algorithm

$$\mathrm{EXP}\left[\begin{array}{l} \left(2.20-(\mathrm{Age}\times 0.0106)+(\mathrm{BSA}\times 0.122\right)\\ -(\mathrm{CYP}2C9\times 0.190)-(\mathrm{VKORC}1AA\times 0.229)-(\mathrm{VKORC}1GA\times 0.636)\\ -(IF(AI=2,0.0742,IF(AI=3,0.118,IF(AI=4,0.120))))\\ +(T_{\mathrm{INR}}\times 0.216)-(\mathrm{Statin}\times 0.0448)-(\mathrm{Amiodarone}\times 0.233)\\ -(\mathrm{Smoke}\times 0.126)+(\mathrm{Diabets}\times 0.135)+(\mathrm{Vit}\_K\times 0.09837) \end{array}\right]$$

**Figure 6:**
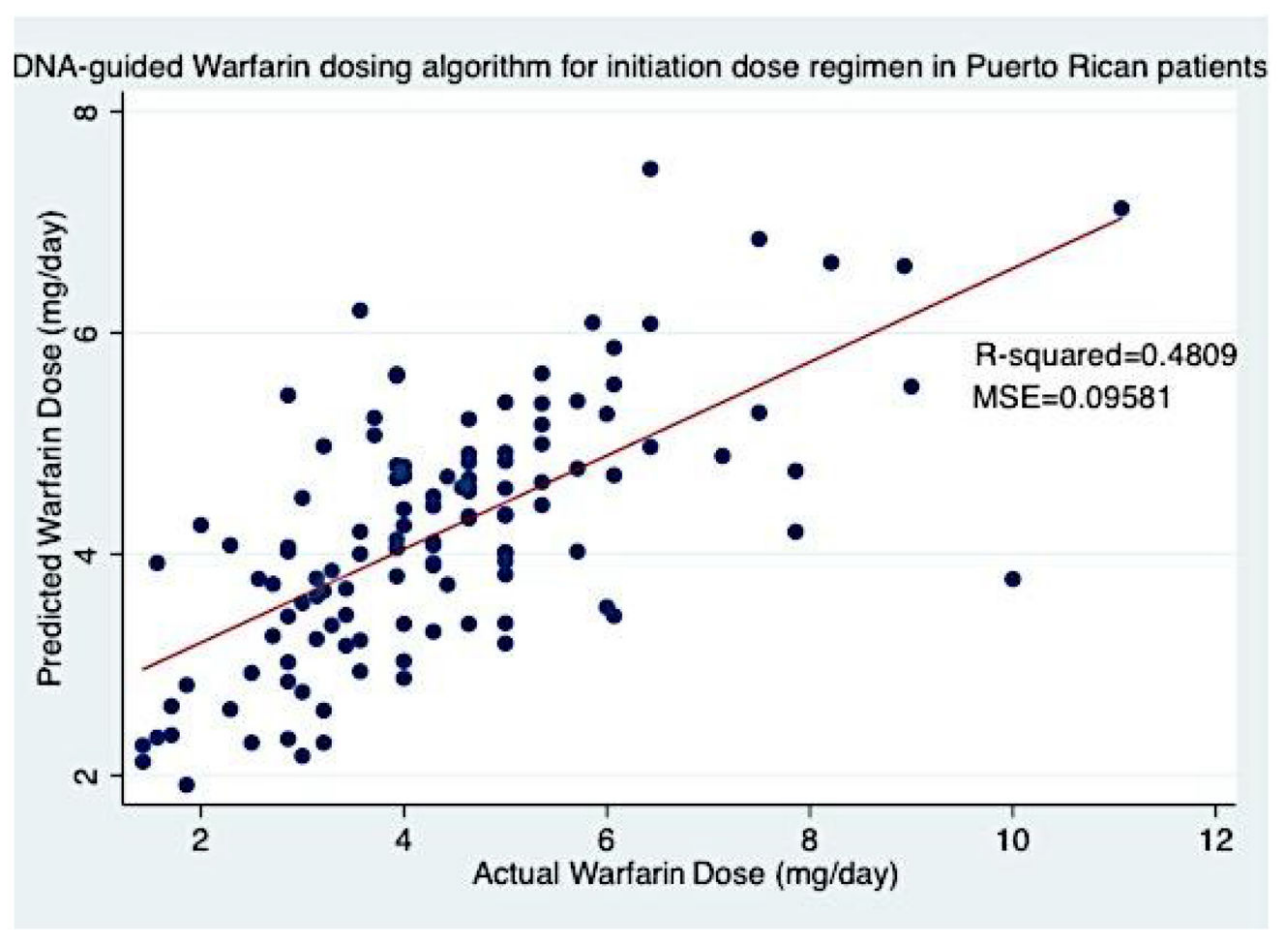
DNA-guided warfarin dosing algorithm for initiation dose regimen in Puerto Rican patients from VACHS, developed by multivariate regression analysis in 121 patients included in our derivation cohort.

Where, EXP is the exponential function; 2.20 is a constant; Age is described in years; BSA is measured in m^2^; CYP2C9 is a carrier code, where 0=Wild-type, 1=single carrier (one mutated allele), 2=double carrier (two mutated alleles); *VKORC1* AA is a carrier code, where 1=AA and 0 otherwise; *VKORC1* GA is a carrier code, where 1=GA and 0 otherwise; Admixture Index (AI) is a carrier code, where, 2=Taino, 3=African, 4=Mixed; Target INR (T_INR_) is a status code for therapeutic range, where 0=therapeutic range of 2–3 and 1= therapeutic range of 2.5–3.5; Statin is 1 if patient uses this class of drug and 0 otherwise; Amiodarone is 1 if patient uses this drug and 0 otherwise; Smoke is 1 if the patient is a smoker and 0 otherwise; Diabetes is 1 if patient presents this condition and 0 otherwise; and Vit_K (Vitamin K intake) is 1 if patients diet is high in vitamin K intake and 0 otherwise. The performance of this pharmacogenetic model is shown in Figure 6.

**Figure 7:**
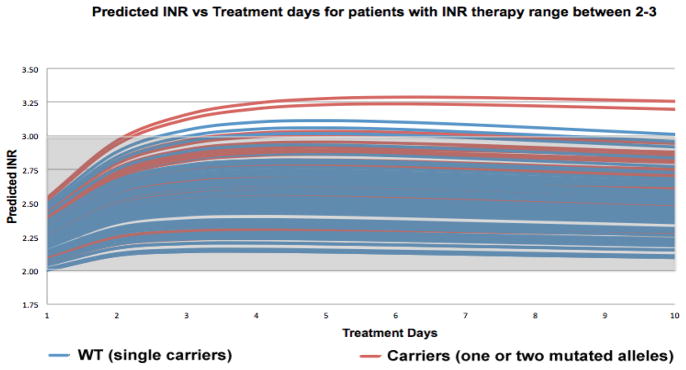
Pharmacokinetic-pharmacodynamic simulations with WinNonlin® software to predict INR response in patients with therapy range of 2–3 (n=114), resulting from the combination of our derived pharmacogenetic-based model (equation 1) for initiation dose regimen, and formulas by Avery et al. [37] to predict the individualized initial dose regimen for patients commencing anticoagulation therapy (patients WPRA001-WPRA171 excluding patients with INR target range of 2.5–3.5).

**Figure 8:**
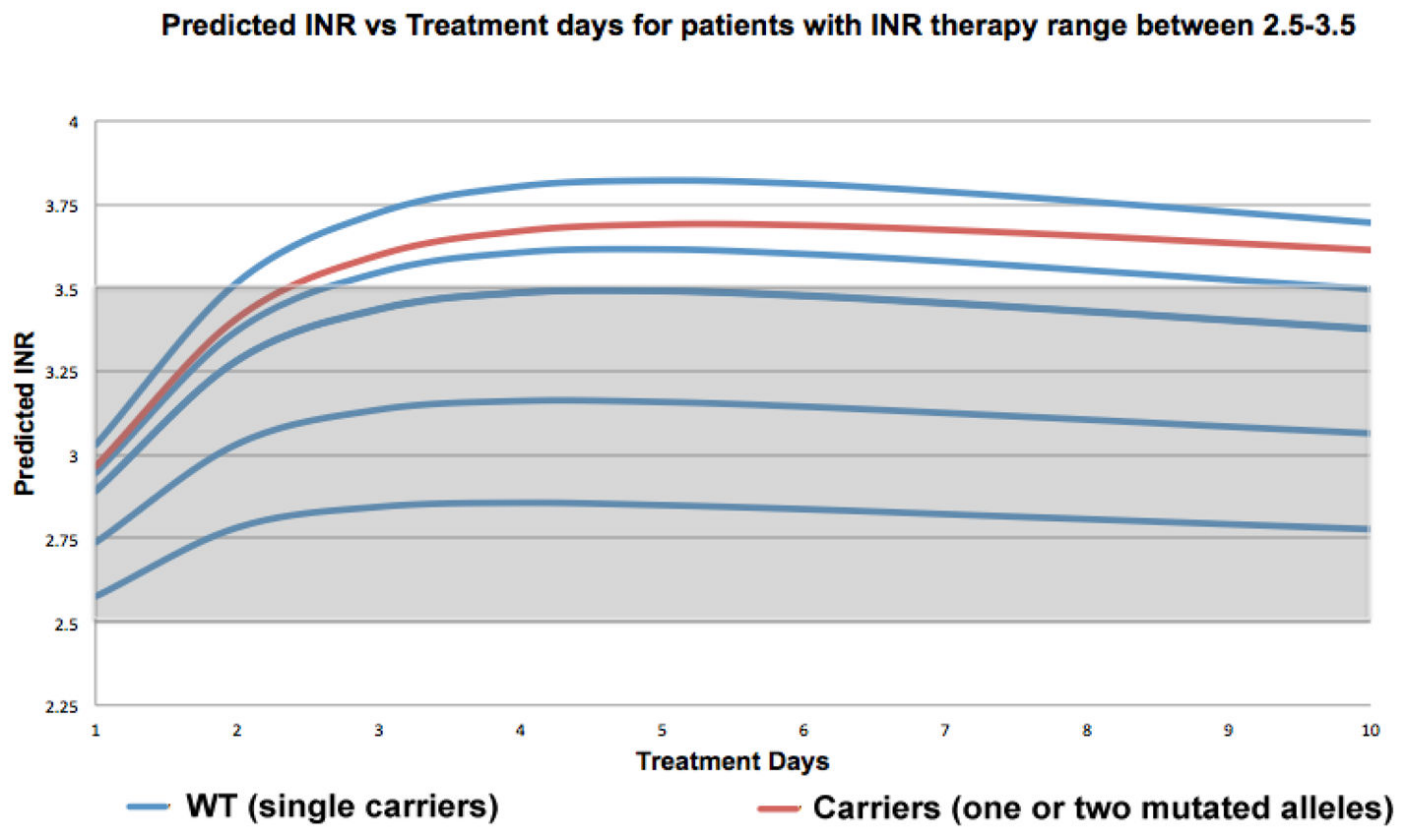
Pharmacokinetic-pharmacodynamic simulations with WinNonlin® software to predict INR response in patients with therapy range of 2–3 (n=114), resulting from the combination of our derived pharmacogenetic-based model (equation 1) for initiation dose regimen, and formulas by Avery et al. [37] to predict the individualized initial dose regimen for patients commencing anticoagulation therapy (patients WPRA001-WPRA171 excluding patients with INR target range of 2.5–3.5).

WinNonlin® pharmacokinetic-pharmacodynamic simulations where carried out using the parameters previously described in our methods section (Table 3) for each of our 121 patients. The first three doses were determined by combining our derived pharmacogenetic-based model (Equation 1) for initiation dose regimen, and formulas by Avery et al. [37] predicted warfarin doses range from; 12.22 to 3.43 mg/day for day 1, 10.49 to 3.22 mg/day for day 2, 8.97 to 2.75 mg/ day for day 3. This enabled us to determine the INR response for each patient and to determine the utility of our pharmacogenetic algorithm (Equation 1) for patients commencing anticoagulation therapy. The resulting 121WinNonlin® simulations were divided into two groups, according to each patient’s therapeutic range, resulting in 114 patients with therapeutic range of 2–3, and 7 patients with therapeutic range of 2.5–3.5 and each group was then combined to establish a collective INR response profile that enabled us to see if there were any differences between WT (single carriers) and carriers (one or two mutated alleles) (Figures 7 and 8).

For patients with an INR therapeutic range of 2–3 (n=114), WinNonlin® simulations resulted in 7 patients over their INR therapeutic range. Of these, 3 patients were WT carriers and 4 represented carriers (one or two mutated alleles). These INR’s over their respective target INR ranged from 3.02 to 3.29. In our second group, patients with an INR therapeutic range of 2.5–3.5 (n=7), WinNonlin® simulations resulted in 3 patients over their INR therapeutic range. Of these, 2 patients were WT carriers and 1 represented carriers (one or two mutated alleles). These INR’s over their respective target INR ranged from 3.61 to 3.82.

Our second multivariate least-squares linear regression focused on the development of a pharmacogenetic-guided algorithm that predicts the log-transformed effective warfarin maintenance dose (Md) (Equation 2). This model incorporated demographic, genetic and a variety of clinical variables which also demonstrated to be the best alternative for the available data, following the lowest mean absolute error criteria. For this linear regression analysis, an additional 7 participants where automatically excluded by Stata®v.12 software because of lack of information regarding INR at day 4 (INR_4). This regression model included 131 patients, the variables included in our algorithm were the following; age (p-value=0.011), Dose 1 (p-value=0.024), Dose 2 (p-value=0.325), Dose 3 (p-value<0.0001), LN_INR_ at day 4, (p-value<0.0001), *CYP2C9* (p-value<0.0001), VKORC1 AA (p-value<0.0001), VKORC1 GA (p-value<0.0001), Admixture Index 2 (Taino) (p-value=0.257), Admixture Index 3 (African) (p-value=0.047), Admixture Index 4 (Mixed) (p-value=0.031), Amiodarone use (p-value=0.223). Overall, the pharmacogenetic model for our maintenance dose regimen contained 10 variables, of these, 7 were statistically significant (p<0.05) and 1 resented a biological plausibility (0.05 ≤ p ≤ 0.15). The regression analysis for this model is summarized in Table 5.

**Equation 2**: Maintenance Dose Algorithm:

$$\mathrm{EXP}\left[\begin{array}{l} \left(1.56-(\mathrm{Age}\times 0.00546)+(D1\times 0.0671\right)\\ -(D2\times 0.0478)+(D3\times 0.156)-({LN}_{\mathrm{INR}4}\times 0.323)\\ -(\mathrm{CYP}2C9\times 0.146)-(\mathrm{VKORC}1AA\times 00.147)-(\mathrm{VKORC}1GA\times 0.333)\\ -(IF(AI-2,0.0593,IF(AI=3,0.111,IF(AI=4,0.108))))\\ -(\mathrm{Amiodarone}\times 0.132) \end{array}\right]$$

**Figure 9:**
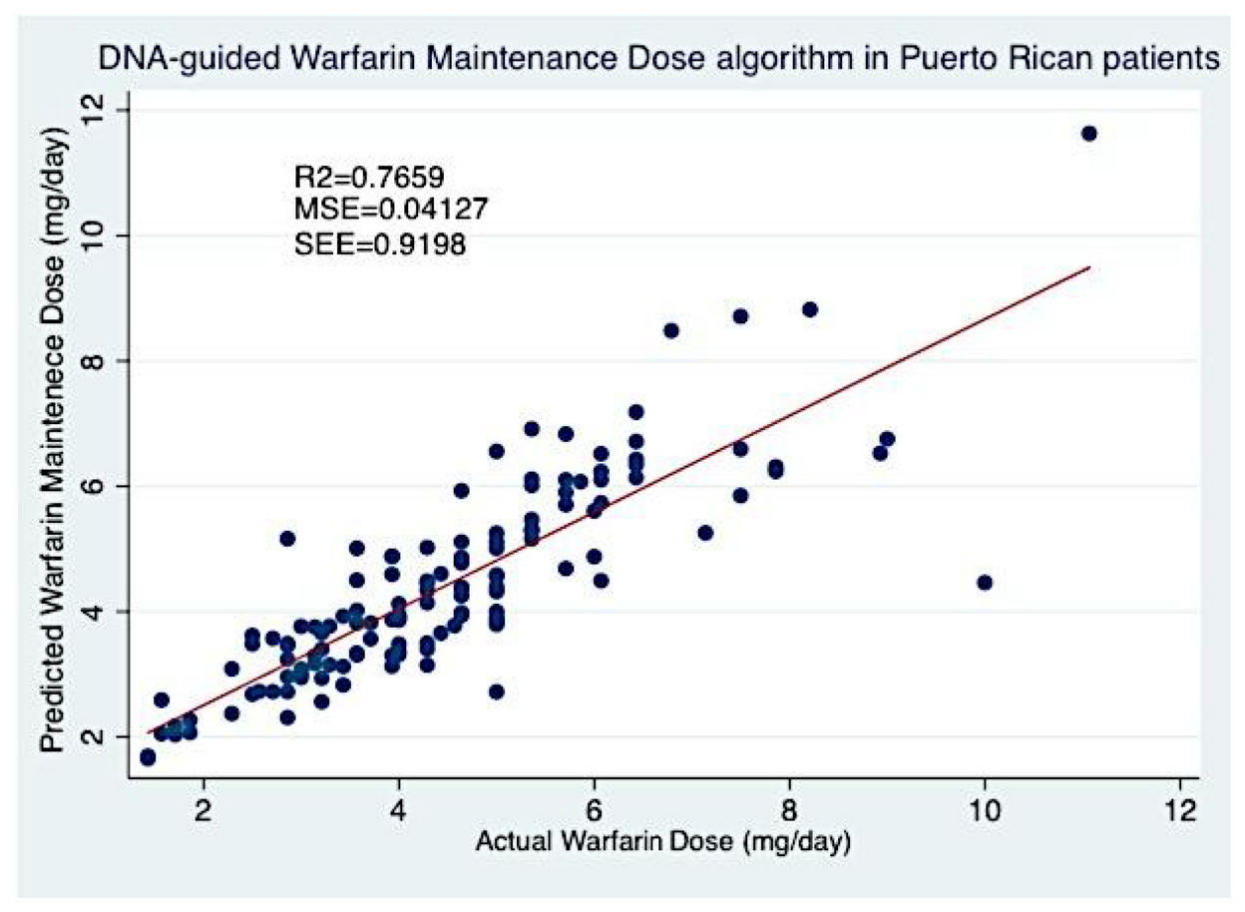
DNA-guided Warfarin maintenance dose algorithm in Puerto Rican patients from VACHS, developed by multivariate regression analysis in 131 patients included in our derivation cohort.

Where, EXP is the exponential function; 1.56 is a constant; Age is described in years; D1 is dose at day 1 (mg); D2 is dose at day 2 (mg); D3 is dose at day 3 (mg); LN_INR4 is the natural logarithm of INR at day 4; CYP2C9 is a carrier code, where 0=Wild-type, 1=single carrier (one mutated allele), 2=double carrier (two mutated alleles); *VKORC1* AA is a carrier code, where 1=AA and 0 otherwise; *VKORC1* GA is a carrier code, where 1=GA and 0 otherwise; Admixture Index (AI) is a carrier code, where, 2=Taino, 3=African, 4=Mixed; Amiodarone is 1 if patient uses this drug and 0 otherwise. The performance of this pharmacogenetic model is shown in Figure 9.

The performance of our pharmacogenetic algorithm was compared among side the International Warfarin Pharmacogenomics Consortium (IWPC) algorithm. Dosages have been divided into three groups; low-dose (≤3 mg/day), intermediate-dose group (>3 and <7 mg/day), and high-dose (≥7 mg/day). The derivation cohort may be found in Tables 6 and 7. This is evidenced by an overall lower MAE for our pharmacogenetic-driven algorithm, than that of the IWPC algorithm (Table 6).

In general, the pharmacogenetic algorithm in Puerto Ricans provided consistently better dose prediction, particularly for patients who required low-doses or intermediate-doses. Interestingly, in 80.1% (n=105) of patients, the absolute difference values were <1 mg/day, (i.e., falling within 20% of the actual dose) which is a well-accepted criteria for accuracy in dose estimation.

## Discussion

To our knowledge, we are the first group to develop a pharmacogenetic-driven warfarin initiation algorithm. Although only 5 out of 12 variables incorporated in our algorithm demonstrated to be statistically significant (p<0.05) and 2 variables demonstrated to be a biological possibility (0.05 ≤ p ≤ 0.15), the remaining variables were included because they are known clinical factors that affect warfarin dosage variability. The variables that are taken into consideration by prescribers when a patient initiates anticoagulation therapy were incorporated. An R^2^=0.4809 was obtained, which is lower when compared to other algorithms [25,27–30], but must point out that the variables included in our algorithm are ones that are available beforehand (age, BSA, *CYP2C9*, *VKORC1*, admixture index, target INR, statin use, amiodarone use, smoker, diabetes and vitamin K intake). However, one limitation of our developed initiation dose algorithm could be the genetic variables, which aren’t immediately available in a clinical setting. Then it was proceeded to combine this developed initiation dose algorithm with pharmacokinetic formulas by Avery et al. [37] to establish a pharmacogenetic-pharmacokinetic model that enabled us to predict the individualized initial doses for patients initiating anticoagulation therapy. When compared, the predicted doses for days 1–3 with the actual doses, the MAE in some cases is extremely high. Please note the intention not to develop a dosage regimen that could predict actual dosages, but one that could enable patients to achieve an INR response in a shorter time frame and one that could reduce the risk of stroke, bleeding or embolism, potentially diminish hospitalization days, which may reduce treatment costs for both patients and health insurances. Even though our R^2^ was low (R^2^=0.4809), WinNonlin® pharmacokinetic-pharmacodynamic simulations demonstrated promising results. For patients with warfarin therapy range between 2–3 (n=114), only 7 (3 WT and 4 carriers) simulations were over their respective therapy range (Figure 7). 93.86% of these simulations were within this therapy range. Even though these over estimates existed, they were between 3.02 and 3.29, which are remarkably close to their therapy range. For our second group, patients with a therapy range between 2.5–3.5 (n=7), 3 simulations (2 WT and 1 carrier) were over their therapy range (Figure 8). 57.14% of these simulations were within their respective therapy range. Over estimations for this group were between 3.61 and 3.82, which are higher than the previous group. There appears to be no difference in over estimates between WT and carriers in both groups. In general, 91.74% (n=121) of all WinNonlin® PK-PD simulations for both groups were within their respective therapy ranges. In the future, the predictability of this algorithm may be improved by evaluating other common polymorphisms in other candidate genes like *CYP4F2, GGCX* and *EPHX1,* or other clinical factors that may be discovered to affect warfarin dosage variability.

The second part of this investigation was focused on the development of a warfarin maintenance dose algorithm with better predictability than any other previously published algorithms [24,27–30,38]. This pharmacogenetic-driven warfarin maintenance dose model contained 10 variables, of these, 7 were statistically significant (p<0.05) and 1 demonstrated to be a biological possibility (0.05 ≤ p ≤ 0.15). This pharmacogenetic Md algorithm achieved a R^2^=0.7659, predicting over ¾ of warfarin maintenance dose variability in this cohort. To our knowledge, this is the highest R^2^ achieved when compared to previously published warfarin pharmacogenetic Md dosing algorithm [24,27–30,38]. This derived pharmacogenetic algorithm resulted in lower MAEs in all three dosing groups (>7 mg/day, ≥3 and ≤ 7 mg/day, <3 mg/day) when compared with the IWPC algorithm (Table 6). This model was compared with the IWPC because it was derived from a large population and has been successively validated in other groups. Overall, the Puerto Rican-oriented pharmacogenetic-driven Md algorithm we developed offered more accurate dose estimates that were considerably closer to the actual doses, when compared to the IWPC algorithm. With the high-dose group, (>7 mg/day) we found that 36.36% achieved an ideal dose and 63.64% is underestimate. However, in this group using the IWPC algorithm, 100% achieved an underestimate. In the intermediate-dose group (≥ 3 and ≤ 7 mg/day), 80.41% were within the ideal-dose group with present derived algorithm vs. 49.49% with the IWPC algorithm. Within this group, present algorithm achieved lower underestimates and lower overestimates when compared to the IWPC algorithm. In the low-dose (<3 mg/day), current methods were able to achieve higher percentages (52.17% vs. 26.09%) in the ideal dose group and lower percentages (47.83% vs. 73.91%) in overestimates (Table 7). Remarkably, the absolute difference values were <1 mg/day in 80.1% (n=105) of our cohort. Unquestionably, the average weekly doses derived from this pharmacogenetic algorithm were higher, when compared to those from the IWPC algorithm (31.00 mg/week vs. 28.33 mg/week). The predictability of this algorithm can also be improved by evaluating other common polymorphisms in other candidate genes like *CYP4F2, GGCX* and *EPHX1.*

The following are recognized as limitations within this study; a single-center study which minimizes generalizability of this findings; the majority of recruited patients were male and only males entered this derivation cohort; a retrospective study design, which can increase chances of overlooking or even missing some events; and finally, the population included in this study represents a typical population that is treated with warfarin (i.e., the elderly), therefore, additional research needs to be conducted on the use of these algorithms in women, younger male adults and children.

## Conclusion

We conducted an exploratory study to establish a warfarin initiation dose regimen. We succeeded by combining the derived pharmacogenetic initiation dose algorithm (Equation 1) and pharmacokinetic formulas by Avery et al. [37]. We developed a novel Puerto Rican-specific pharmacogenetic-pharmacokinetic warfarin initiation dosage regimen. Although it’s predictive value is of 48.09%, WinNonlin® pharmacokinetic-pharmacodynamic simulation demonstrated astonishing findings. It was determined that 91.74% (n=121) of all simulations were within their respective therapy ranges.

Also, a pharmacogenetic warfarin maintenance dose (Md) algorithm (equation 2) was developed with a predictive value of 76.59% and compared its predictability, with the algorithm published by the International Warfarin Pharmacogenomics Consortium (IWPC). The IWPC performed inadequately when applied to current Puerto Rican patient cohort, with significant scatter, low correlation coefficient (R^2^=0.0058) and higher MAEs (Table 6). This developed pharmacogenetic warfarin maintenance dose (Md) algorithm (Equation 2) proved to have a higher predictability within the Puerto Rican population, when compared to the IWPC algorithm (Table 7).

Present findings suggest that it is necessary to expand this project to several other hospitals, in order to amplify the sample population for both of these derivation cohorts, which could enable us to include females in the study and expand the age range. Lastly, after validating the derived models, further work includes to apply them in prospective studies. This will enable to fulfill demands regarding prospective studies that incorporate algorithms and compare adverse event rates between pharmacogenetic-guided and standard dosing of warfarin-based anticoagulation.

## Figures and Tables

**Table 1:** Advantages and disadvantages of warfarin therapy.

**Advantages** Proven efficacy in prevention and treatment of thromboembolic disease Oral Dosing Quick reversal of anticoagulant effect Easily available antidote
**Disadvantages** Narrow therapeutic range Requires monitoring with frequent blood tests Frequent INR fluctuations with a high percentage of readings outside the therapeutic range Requires a high level of patient compliance especially with monitoring Risks of spontaneous bleeding including hemorrhagic stroke Slow onset of the full therapeutic effect Numerous food and drug interactions High rates of treatment discontinuation

**Table 2:** *CYP2C9* and *VKORC1* variants detected with HILOMet Warfarin system on Luminex^®^ 100× Map™ technology. Effects on enzymatic activity are also depicted.

*CYP2C9*			*VKORC1*		
Allelic Variant	Change to Protein	Activity	Allelic Variant	Change to Protein	Activity
*1(WT)	Reference	Normal	WT^#^	Reference	Normal
*2 (430C>T)	Arg144Cys	Decreased	−1639 G>A	Promoter	Deficient
*3 (1075A>C)	Ile359Leu	Null	+85 G>T	Val29Leu	Null
*4 (1076T>C)	Ile359Tyr	Decreased	+121 G>T	Ala41Ser	Null
*5 (1080C>G)	Asp360Glu	Decreased	+134 T>C	Val45Ala	Null
*6 (818delA)	Frameshift	Null	+172 A>G	Arg58Gly	Null
			+1331 G>A	Val66Met	Null
			+3487 T>G	Leu28Arg	Null

Wild-types are assigned as a result of the absence of other SNPs.

**Table 3:** Summary of parameters values applied to WinNonlin® simulations [34].

Parameter	Value	Genotype
*K_a_* (hr^−1^)	1.17	
*Vd* (liters)	8.0	
R_0_ (INR units)	2.5	
R_in_ (INR units)	1.5	
IC_50_ (mg/L)	1.5	
*k_e_* (hr^−1^)	0.0189	*1/*1
	0.0158	*1/*2
	0.0132	*1/*n
	0.0130	*2/*2
	0.009	*2/n
	0.0075	n**/**n
Where n= *3, *5 or *6		

K_a_= Absorption rate constant, Vd= Volume of distribution, R_0_= Rate of elimination, R_in_= Input rate, IC_50_= half maximal inhibitory concentration, k_e_= Elimination rate constant. (These parameters were taken from the literature [34], except k_e_ that varies according to individual genotype).

**Table 4:** Summary of Attributes for Equation 1 of the Pharmacogenetic Warfarin Dosing Initiation Prediction in Puerto Ricans using the Derivation Cohort from the VACHS.

Variables_a_		Partial regression coefficient	SE	R^2^ after entry	p-value
Age		−0.0106	0.003	0.0564	0.003
BSA		0.131	0.158	0.0658	0.406
*CYP2C9*		−0.192	0.048	0.1421	0.000
*VKORC1*AA		−0.227	0.067	0.1538	0.001
*VKORC1* GA		−0.635	0.092	0.4088	0.000
Admixture Index	2	−0.0780	0.082	0.4210	0.345
	3	−0.116	0.088		0.192
	4	−0.124	0.079		0.119
Target INR		0.215	0.126	0.4317	0.093
Statins		−0.0491	0.060	0.4398	0.415
Amiodarone		−0.237	0.171	0.4514	0.168
Smoke		−0.118	0.130	0.4568	0.368
Diabetes		0.137	0.066	0.4741	0.040
Vitamin K		0.0943	0.090	0.4809	0.297

Constant value of the model equation is 2.189 (SE: 0.457).

a Variables are listed in the order they were incorporated into the model using stepwise regression analysis.

**Table 5:** Summary of attributes for equation 2 of the pharmacogenetic warfarin Md prediction in Puerto Ricans using the derivation cohort from the VACHS.

Variables^a^		Partial regression coefficient	SE	R^2^ after entry	p-value
Age		−0.00546	0.002	0.0564	0.011
D1		0.0671	0.029	0.4844	0.024
D2		−0.0478	0.048	0.5288	0.325
D3		0.156	0.041	0.5922	0.000
LN_INR4		−0.323	0.053	0.6757	0.000
CYP2C9		−0.146	0.032	0.7015	0.000
VKAA		−0.147	0.041	0.7053	0.000
VKGA		−0.333	0.065	0.7503	0.000
Admixture Index	2	−0.0593	0.052	0.7629	0.257
	3	−0.111	0.055		0.047
	4	−0.108	0.049		0.031
Amiodarone		−0.132	0.108	0.7659	0.223

Constant value of the model equation is 1.565 (SE: 0.196).

a Variables are listed in the order they were incorporated into the model using stepwise regression analysis.

**Table 6:** Predicted warfarin daily doses (mg/day) with Puerto Rican population, using our derived pharmacogenetic algorithm and IWPC algorithms as compared to the actual doses of warfarin for the therapeutic effect in patients requiring High- Doses (>7 mg/day), Intermediate-Doses (≥ 3 and ≤ 7 mg/day) or Low-Doses (<3 mg/day) in our study cohort of 131 patients at VACHS anticoagulation clinic.

Prediction Model	IWPC algorithm	Puerto Rican Algorithm
**High-Doses (>7 mg/day)**		
MAE (mg/day) 95% CI	4.68 (5.57-3.78)	1.83 (2.64-1.03)
R^2^ (%)	2.34	18.01
*p-*value	0.0525	
**Intermediate-Doses (≥ 3 and ≤ 7 mg/day)**		
MAE (mg/day) 95% CI	1.18 (1.33-1.03)	0.52 (0.61-0.42)
R^2^ (%)	0.58	66.74
*p-*value	1.67×10^−10^	
**Low-Doses (<3 mg/day)**		
MAE (mg/day) 95% CI	1.92 (2.28-1.57)	0.53 (0.74-0.33)
R^2^ (%)	1.75	50.24
*p-*value	0.0259	

**Table 7:** Percentage (%) of patients in the study cohort with an ideal, underestimated, or overestimated dose of warfarin, as estimated with the IWPC pharmacogenetic algorithm and our pharmacogenetic algorithm (equation 2) in patients requiring low, intermediate, or high doses of warfarin for a therapeutic effect^a^.

Model	No. of Patients	Ideal Dose (%)	Underestimate (%)	Overestimate (%)
**High-Doses (>7 mg/day)**				
IWPC algorithm	11	0	100.00	0
Puerto Rican Algorithm		45.45	54.55	0
**Intermediate- Doses (≥3 and ≤7 mg/day)**				
IWPC algorithm	97	33.00	43.33	22.68
Puerto Rican Algorithm		79.38	12.37	8.25
**Low-Doses (<3 mg/day)**				
IWPC algorithm	23	13.04	4.35	82.61
Puerto Rican Algorithm		52.17	0	47.83

a The ideal dose was defined as a predicted dose that was within 20% of the actual stable therapeutic dose of warfarin, underestimation was defined as a predicted dose that was at least 20% lower than the actual dose, and overestimation was defined as a predicted dose that was at least 20% higher than the actual dose.

## References

[1] Johnson JA, Gong L, Whirl-Carrillo M, Gage BF, Scott SA (2011). Clinical Pharmacogenetics Implementation Consortium Guidelines for CYP2C9 and VKORC1 Genotypes and Warfarin Dosing. J Clin Pharmcol Ther.

[2] Mavrakanas T, Bounameaux H (2011). The potential role of new oral anticoagulants in the prevention and treatment of thromboembolism. Pharmacol Ther.

[3] Bristol Myers Squibb Pharma Company (2011). Coumadin (Warfarin Sodium) Label.

[R4] 4Drug Topics (2011) 2010 Top 200 generic drugs by total prescriptions 1–3.

[5] IMS Institute for Health Care Informatics (2012). The Use of Medicines in the United States.

[6] Wysowski DK, Nourjah P, Swartz L (2007). Bleeding complications with warfarin use: a prevalent adverse effect resulting in regulatory action. Arch Intern Med.

[7] Oake N, Jennings A, Forster AJ, Fergusson D, Doucette S (2008). Anticoagulation intensity and outcomes among patients prescribed oral anticoagulant therapy: a systematic review and meta-analysis. CMAJ.

[8] Takahashi H, Echizen H (2001). Pharmacogenetics of warfarin elimination and its clinical implications. Clin Pharmacokinet.

[9] O’Reilly RA (1987). Warfarin Metabolism and Drug-Drug Interactions. The New Dimensions of Warfarin Prophylaxis.

[10] Lewis RJ, Trager WF (1970). Warfarin metabolism in man: identification of metabolites in urine. J Clin Invest.

[11] Miners JO, Birkett DJ (1998). Cytochrome P4502C9: an enzyme of major importance in human drug metabolism. Br J Clin Pharmacol.

[12] Wadelius M, Chen LY, Eriksson N, Bumpstead S, Ghori J (2007). Association of warfarin dose with genes involved in its action and metabolism. Hum Genet.

[13] Choonara IA, Malia RG, Haynes BP, Hay CR, Cholerton S (1988). The relationship between inhibition of vitamin K1 2,3-epoxide reductase and reduction of clotting factor activity with warfarin. Br J Clin Pharmacol.

[14] Stenflo J, Fernlund P, Egan W, Roepstorff P (1974). Vitamin K dependent modifications of glutamic acid residues in prothrombin. Proc Natl Acad Sci U S A.

[15] Kimmel SE (2008). Warfarin therapy: in need of improvement after all these years. Expert Opin Pharmacother.

[16] Food and Drug Administration (2007). FDA approves updated warfarin (Coumadin) prescribing information.

[17] Wadelius M, Chen LY, Downes K, Ghori J, Hunt S (2005). Common VKORC1 and GGCX polymorphisms associated with warfarin dose. Pharmacogenomics J.

[18] Schwarz UI, Ritchie MD, Bradford Y, Li C, Dudek SM (2008). Genetic determinants of response to warfarin during initial anticoagulation. N Engl J Med.

[19] Scott SA, Khasawneh R, Peter I, Kornreich R, Desnick RJ (2010). Combined CYP2C9, VKORC1 and CYP4F2 frequencies among racial and ethnic groups. Pharmacogenomics.

[20] Krishna Kumar D, Shewade DG, Loriot MA, Beaune P, Balachander J (2013). Effect of CYP2C9, VKORC1, CYP4F2 and GGCX genetic variants on warfarin maintenance dose and explicating a new pharmacogenetic algorithm in South Indian population. Eur J Clin Pharmacol.

[21] McDonald MG, Rieder MJ, Nakano M, Hsia CK, Rettie AE (2009). CYP4F2 is a vitamin K1 oxidase: An explanation for altered warfarin dose in carriers of the V433M variant. Mol Pharmacol.

[22] Mitchell C, Gregersen N, Krause A (2011). Novel CYP2C9 and VKORC1 gene variants associated with warfarin dosage variability in the South African black population. Pharmacogenomics.

[R23] 23CYP2C9 allele nomenclature.

[24] Liu Y, Jeong H, Takahashi H, Drozda K, Patel SR (2012). Decreased warfarin clearance associated with the CYP2C9 R150H (*8) polymorphism. Clin Pharmacol Ther.

[25] Wei M, Ye F, Xie D, Zhu Y, Zhu J (2012). A new algorithm to predict warfarin dose from polymorphisms of CYP4F2, CYP2C9 and VKORC1 and clinical variables: derivation in Han Chinese patients with non valvular atrial fibrillation. Thromb Haemost.

[26] Michaud V, Vanier MC, Brouillette D, Roy D, Verret L (2008). Combination of phenotype assessments and CYP2C9-VKORC1 polymorphisms in the determination of warfarin dose requirements in heavily medicated patients. Clin Pharmacol Ther.

[27] Gage BF (2006). Pharmacogenetics-based coumarin therapy. Hematology Am Soc Hematol Educ Program.

[28] Klein TE, Altman RB, Eriksson N, Gage BF, International Warfarin Pharmacogenetics Consortium (2009). Estimation of the warfarin dose with clinical and pharmacogenetic data. N Engl J Med.

[29] Wu AH, Wang P, Smith A, Haller C, Drake K (2008). Dosing algorithm for warfarin using CYP2C9 and VKORC1 genotyping from a multi-ethnic population: comparison with other equations. Pharmacogenomics.

[30] Zhu Y, Shennan M, Reynolds KK, Johnson NA, Herrnberger MR (2007). Estimation of warfarin maintenance dose based on VKORC1 (−1639 G>A) and CYP2C9 genotypes. Clin Chem.

[31] Lenzini P, Wadelius M, Kimmel S, Anderson JL, Jorgensen AL (2010). Integration of genetic, clinical, and INR data to refine warfarin dosing. Clin Pharmacol Ther.

[32] Horne BD, Lenzini PA, Wadelius M, Jorgensen AL, Kimmel SE (2012). Pharmacogenetic Warfarin Dose Refinements Remain Significantly Influenced by Genetic Factors after One Week of Therapy. Thromb Haemost.

[33] Cavallari LH, Momary KM, Patel SR, Shapiro NL, Nutescu E (2011). Pharmacogenomics of warfarin dose requirements in Hispanics. Blood Cells Mol Dis.

[34] Gong IY, Tirona RG, Schwarz UI, Crown N, Dresser GK (2011). Prospective evaluation of a pharmacogenetics-guided warfarin loading and maintenance dose regimen for initiation of therapy. Blood.

[35] Willey VJ, Bullano MF, Hauch O, Reynolds M, Wygant G (2004). Management patterns and outcomes of patients with venous thromboembolism in the usual community practice setting. Clin Ther.

[36] Garcia DA, Lopes RD, Hylek EM (2010). New-onset atrial fibrillation and warfarin initiation: high risk periods and implications for new antithrombotic drugs. Thromb Haemost.

[37] Avery PJ, Jorgensen A, Hamberg AK, Wadelius M, Pirmohameh M (2011). A proposal for an individualized pharmacogenetic-based warfarin initiation dose regimen for patients commencing anticoagulation therapy. Clin Pharmacol Ther.

[38] Ramos AS (2011). Pharmacogenetic-driven Warfarin Dosing Algorithm in Puerto Ricans. MSc Thesis.

